# Effectiveness of physiotherapy interventions for injury in ballet dancers: A systematic review

**DOI:** 10.1371/journal.pone.0253437

**Published:** 2021-06-24

**Authors:** Marlena Skwiot, Zbigniew Śliwiński, Arkadiusz Żurawski, Grzegorz Śliwiński

**Affiliations:** 1 Faculty of Health Sciences, Jan Kochanowski University in Kielce, Kielce, Poland; 2 Institute of Biomedical Engineering, Faculty of Electrical and Computer Engineering, TU Dresden, Dresden, Germany; The Wingate College of Physical Education and Sports Sciences at the Wingate Institute, IL, ISRAEL

## Abstract

**Background:**

The unique repetitive nature of ballet dancing, which often involves transgressing endurance limits of anatomical structures, makes dancers prone to injury. The following systematic review aims to assess the effectiveness of physiotherapy interventions in the treatment of injuries in ballet dancers.

**Methods:**

The review was performed in line with the PRISMA statement on preferred reporting items for systematic reviews and meta-analyses. Six electronic databases (PubMed, Ovid Embase, Cochrane, Medline, PEDro, Google Scholar) were queried. The study populations comprised active ballet dancers and/or ballet school attendees with acute and chronic injuries and those with persistent pain. There were no restrictions regarding age, sex, ethnicity or nationality. The Modified McMaster Critical Review Form for quantitative studies was used to assess the methodological quality of the studies reviewed in accordance with the relevant guidelines.

**Results:**

Out of the total of 687 articles subjected to the review, 10 met the inclusion criteria. Diverse physiotherapeutic interventions were described and effectiveness was assessed using different parameters and measurements. Overall, the results indicate that physiotherapy interventions in ballet dancers exert a positive effect on a number of indices, including pain, ROM and functional status.

**Conclusions:**

Due to the small amount of evidence confirming the effectiveness of physiotherapeutic interventions in ballet dancers after injuries and methodological uncertainties, it is recommended to improve the quality of prospective studies.

## Introduction

Ballet is high-performance dancing that requires a high level of technical skills [1]. Particular demands are placed on flexibility and strength as well as on body aesthetics [2, 3]. The unique repetitive nature of ballet dancing, which often involves transgressing endurance limits of anatomical structures, makes dancers prone to injury [4]. The need to understand the mechanisms of injury in dancers is a considerable challenge on account of methodological limitations regarding injuries and characteristics of the population of dancers [3, 5].

Previous reports have noted the presence of injuries and some risk factors underlying these injuries in ballet dancers. In these studies, acute injuries were observed rarely and were usually associated with loss of balance during practice or a performance. At the same time, overload-related injuries were common, mostly affecting the lower limbs and the lower back (lumbosacral spine) [3, 6–8]. It has been found that ballet dancers may be at risk of low back pain or injury independent of gender, age or level of mastery [9]. Important risk factors for injuries included intensity of training [10, 11], poor control of lumbosacral complex motion, inadequate lower limb strength, poor oxygen endurance [12–14], and lifting in male dancers [15]. Due to the lack of high-quality research, consensus on risk factors for musculoskeletal injuries in dancers remains difficult. There is a need for high-quality prospective studies exploring the multifactorial relationship between risk factors and dance injuries [16].

Some studies have focused mainly on orthopaedic surgery interventions. Foot surgery has often been described [17–20] in relation to such injuries as metatarsal fractures, ankle impingement or tendinopathies. Surgical vs conservative management of fractures in dancers has also been described [21–23]. The hip joints in dancers have increasingly been a topic of interest for researchers, owing to their extreme mobility, with regard to biomechanics and treatment on account of both joint and muscle pathology. In a systematic review, Weber et al. concluded that appropriate surgical indications and good hip surgery techniques can prevent a premature end of career in dancers [24], while Nolton et al. reported on the need to introduce early and comprehensive work-up and management of snapping hip syndrome (SHS) [25].

Treatment of musculoskeletal conditions in ballet dancers has included physiotherapy intervention, including shockwave therapy [27, 28], manual therapy [29–31], stability exercises [32, 33], home exercise programs [31, 33–35] and stretching [36] as well as dry needling and acupuncture [31, 35]. Research has pointed to a level of effectiveness of physiotherapy in reducing pain and improving function [27–36]. However, the evidence base is limited and no systematic reviews to date have analyzed the effectiveness of physiotherapy in the treatment of musculoskeletal dysfunctions in dancers. Accordingly, this review aims to assess the effectiveness of physiotherapeutic interventions in the treatment of injuries in ballet dancers.

## Methods

### Search strategy

The review was performed in line with the PRISMA statement on preferred reporting items for systematic reviews and meta-analyses and is compatible with the PRISMA checklist (S1 File). The population-intervention-comparator-outcome (PICO) format was used for the search strategy, where the search terms and limits were associated with ballet dancers (population of interest) and physiotherapy (intervention).

To test the search strategy, the databases were searched independently by two reviewers. The search results were then compared to ensure search consistency. In cases where the results were different, the two reviewers discussed these differences together, and any conflicts were resolved by discussion. Only after achieving search consistency did the reviewers begin their formal queries of the databases. A comprehensive search strategy was developed and six electronic databases (PubMed, Ovid Embase, Cochrane, Medline, PEDro and Google Scholar) were queried between March 26 and March 28, 2020. Only articles in English were taken into account.

As there had been no previous systematic reports, no date limitation was applied. The following MESH headings were used: dancer, ballet, ballet dancer, classical dancer, pain, and physical therapy. The complete data base search strategy is described in S2 File.

### Study design

All types of quantitative study design were eligible for inclusion, including randomized controlled trials (RCT), controlled clinical trials (CCT), case studies, pre-post cohort studies and quasi-experimental studies. The inclusion criteria for the PICO format are presented below.

#### Population

Studies of interest were those investigating active ballet dancers and/or ballet school attendees with acute and chronic injuries and persistent pain. There were no restrictions regarding age, sex, ethnicity or nationality. Dancers experiencing a break from activity due to injury were included, but studies of retired dancers, dance instructors as well as those whose dominant style was not ballet dancing were excluded. Studies were also excluded if the study group was not defined precisely enough and was described using such general terms as “dancers” or “professional dancers”.

#### Intervention

As physiotherapy is usually concerned with comprehensive interventions, the review was not limited to a particular type of interventions and intervention parameters were not specified.

Physiotherapy interventions included, but were not limited to, kinesiotherapy, physical therapy, manual therapy, needle therapy and education. Studies where the intervention was limited to orthopaedic procedures or pharmacotherapy were excluded. Comprehensive interventions (physiotherapy combined with other modalities) were allowed.

#### Comparator

Admissible comparator interventions were non-intervention control and usual care.

#### Outcome

In view of the multidimensional nature of pain, differences in injury sites, nature of injury and a diversity of outcomes representing effects of physiotherapy interventions the queries were not limited with regard to any specific outcomes, with outcomes of interest including pain, ROM (range of motion), functional status and quality of life.

### Study selection

The results of the literature searches were transferred to the reference management software EndNote X9 to allow sorting of the identified studies. Duplicate results were removed. The studies were then accepted or excluded by analyzing the title and summary according to the PICO criteria. The full texts of accepted papers were analyzed by two independent reviewers to determine compatibility with the PICO criteria. Any disputes were resolved by discussion.

### Methodological quality

The accepted studies were independently assessed and classified according to “category of intervention” of the National Health and Medical Research Council (NHMRC) hierarchy of evidence by two reviewers. The Modified McMaster Critical Review Form [26] for quantitative studies was used to assess the methodological quality of the studies reviewed in accordance with the relevant guidelines. The tool assessed the following eight domains: study purpose; literature review; study design (all experimental designs); sample (description of subjects, justification of sample size, ethics, consent); outcomes (reliability and validity, outcome areas and outcome measures employed); intervention (description, contamination, co-intervention); results (statistical significance and clinical importance, analysis methods, drop-outs); conclusions and clinical implications (limitations and bias). Individual elements were rated as “Yes”, “No”, “Not addressed” or “NA–not applicable”. Answers “Yes” were awarded a mark of 1, “No” and “Not addressed”, a mark of 0; if the answer was “NA”, the total score was altered appropriately. Depending on the study design and particular specific items, the maximum total score was 14. Each study was rated independently by each reviewer and any disputes were resolved by discussion.

### Summary of results

A meta-analysis was not performed in view of heterogeneity of the studies. A descriptive summary was carried out instead.

## Results

**Fig 1:**
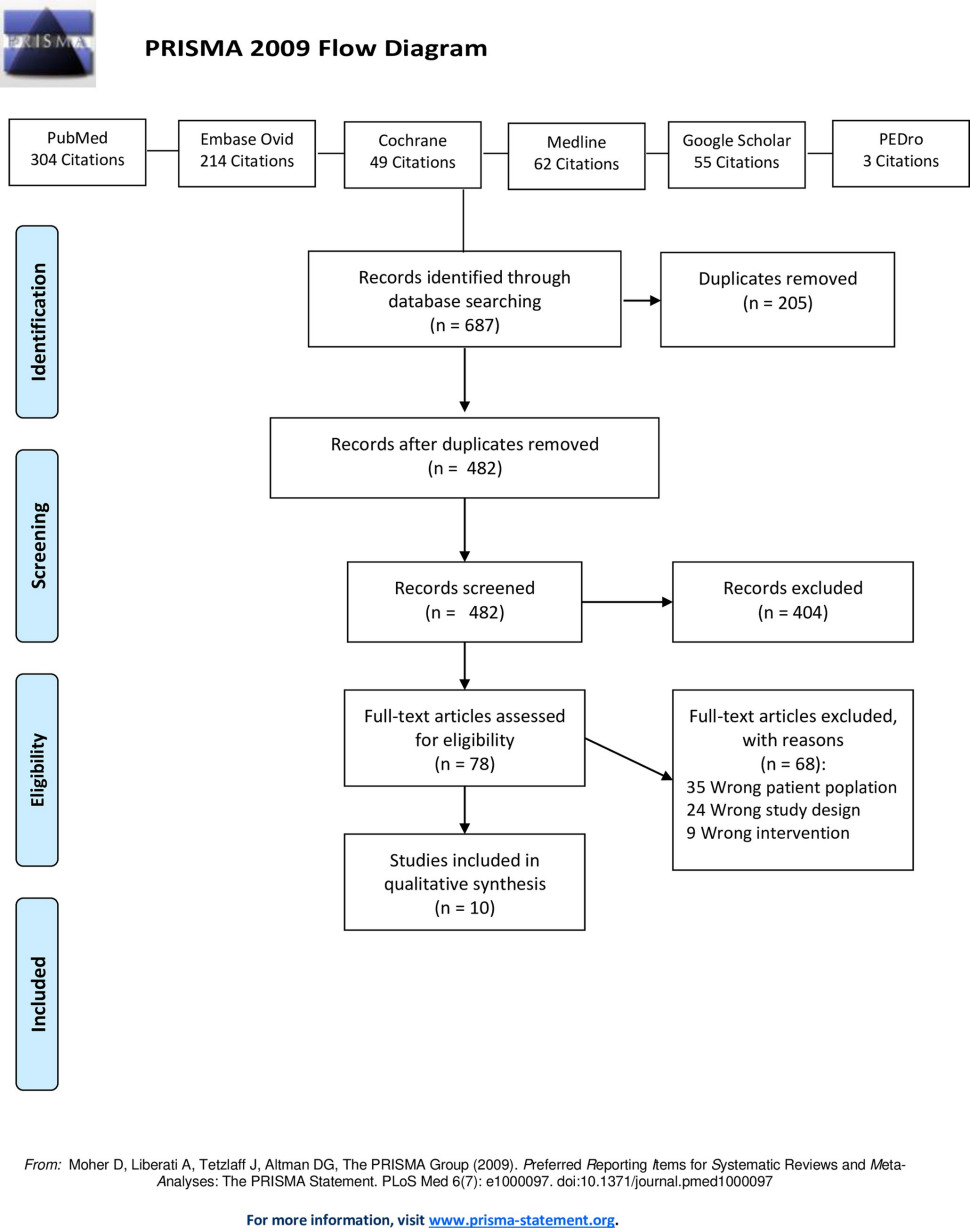
PRISMA block diagram.

The literature search yielded 687 results, including 205 duplicates, which were subsequently removed. Analysis of the 482 titles and summaries resulted in the exclusion of 404 items. The remaining 78 articles were analyzed in detail, with 68 subsequently excluded (35 on account of the study population not meeting the inclusion criteria, 24 on account of an inappropriate study design, and 9 on account of an inappropriate intervention). A total of ten articles were compatible with all inclusion criteria and were consequently included in the study. The relevant PRISMA block diagram is presented in Fig 1.

### Risk of bias

**Fig 2:**
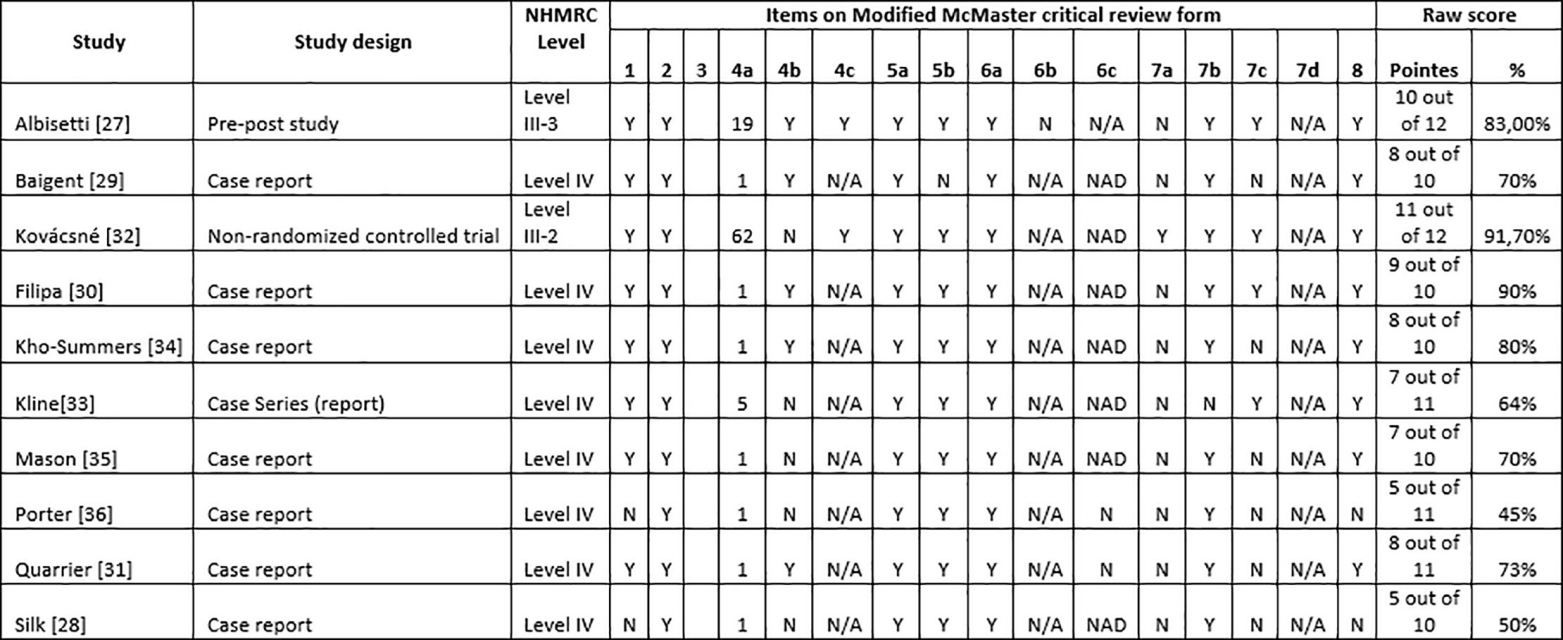
Items on Modified McMaster Critical Review form.

Fig 2 summarizes NHMRC levels of evidence and scores assigned to the 10 studies according to the McMaster Quantitative Critical Appraisal Tool [26]. Study designs comprised 1 pre-post study [27], 1 non-randomized case-control study [32], 1 case series [33] and 7 case reports [28–31, 34–36]. The two highest critical appraisal scores of 91.7% and 90% were assigned to the studies by Kovácsné and Filipa et al., respectively, while the lowest score was assigned to the study by Porter et al. (45%), which indicates the lowest quality of that study among the 10 studies. All studies appropriately presented significant basic information and provided a rationale.

The chief methodological shortcoming in the 10 studies was the lack of randomization as 8 of the 10 works were reported case series or case reports. There was no rationale for the sample size in 5 out of the 10 studies [28, 32, 33, 35, 36], while only one study used statistical analysis [32]. Additionally, ethical approval was mentioned in only two studies [27, 32].

### Study characteristics

Table 1 summarizes characteristics of the studies. The present systematic review identified various models of research. The studies were carried out in the USA [30, 31, 33–36], Italy [27], New Zealand [29], Hungary [32] and the UK [28]. The papers were published between 1998 and 2018. All studies investigated the effect of physiotherapy interventions on pain reduction and improvement in musculoskeletal function in ballet dancers.

**Table 1:** Characteristics of the studies.

Follow up	2,2 years follow up, 3-5 weeks return to dance	7 months	3 months	6 months	5 months	1,5 months	3 months	9 months	6 years	1 year
**Outcome addressed**	MRI, X-ray, CT	NRS, ROM, posture and gait analise, X-ray	VAS, LL-test, Core-test, posture analise	NRS, ROM, RTD, DFOS, SL balance with eyes closed test, manual muscle tests, Airplane Test, the Topple Test, PedsQl, MRI, Beighton scale X-ray	NRS, SLR, Sahrmann	NRS, ROM, SLR, Core-test, PSFS	NRS, ROM, SLR, Thomas, Ober, FLS squat, deep squat, posture and gait analise, MRI, X-ray	ROM, posture and gait analise, MRI, X-ray, USG	ROM, Thomas, Ober, Patrick, X-ray, MRI, Kendall and McCreary’s manual muscle test grading and guidelines, specific manual muscle tests of the left hip, palpation of the groin, supine to sit test	X-ray, CT
**Comparator/control**			Tree-months spine prevention programme			The dynamic sling exercises were performed on a Redcord System, with a floot mat placed under the device fot safety. These participants did approximately 20 minutes of home exercises one time per week and exercised using the dynamic sling system fot 25 to 30 minutes two times a week for 6 weeks.				
**Co-intervention**		Chiropractic		Malleo Train (active stabilizer)				Walking boot, surgical intervention	Medications, psychological counsulting, chiropractic, arthroscopy, surgical intervention	
**Intervention**	18: ESWT; 1: US + EMF	Soft tissue therapy: isometric contraction of bilateral psoas major muscle; concentric muscle stretches prescribed for piriformis, psoas major and vastus medialis, thermotherapy (alternate heat and ice)	Tree-months spine prevention program. The program consists of three units: 1st–2nd months: raising awareness to and automatizing the correct posture, core muscle strengthening and stretching exercises to facilitate sufficient muscle balance, exercises to improve lumbar motor control ability. The 3rd month: the muscle balance and lumbar motor control exercises include dance-specific exercise material	Therapeutic exercises, therapeutic activity, modalites, neuromuscular reeducation, manual therapy, cryotherapy, e-stim, pre-mod. The intervention was divided into 4 levels: - acute (1-4 weeks), - early RTD (5-10 weeks), - RTD (11-15 weeks) - end-stage RTD (16-20 weeks). The intervention concerned: pain reduction, increase ROM, increase foot/ankle strength, increase hip/knee strength, increase lumbopelvic control, increase lower extremity flexibility, improving balance, increase cardiovascular endurance, improving closed kinetic chain functional mobility	Education about faulty postures and movement impairments, supervised practice of walking and dance, home exercise program. Education for functional activities included corrections for impairments in standing and sitting. Corrections for gait included instructions to lift the heel sooner after midstance. The home exercise program consisted of a variety of exercises to improve muscle performance and precision of hip flexion and extension.	Lumbar stabilization, home exercise program. The HEP consisted of approximately 20 minutes of the same exercises (bridge, plank, side plank) used for strength and endurance testing, three times a week for 6 weeks.	Dry needling, home exercise program. Treatment with dry needling at two separate visits, with 48 hours between treatment sessions. In both sessions DN was performed to target the palpable areas consistent with MTPs in the right gastrocnemius, soleus, and distal popliteus at the attachment to the medial tibia. Home exercise program after first visit: standing gastrocnemius and standing soleus stretches - each stretch 2 to 3 times, statically holding for a minimum of 30 seconds, repeated 3 to 4 times daily. Progress in home exercise program: single leg squat with contralateral isometric hip abduction with instructions to progress to single leg squat focusing on pelvic stability and proper knee alignment and hip abduction with resisted side stepping using a resistance band.	Stretching, eccentric strengthening (before surgical intervention); continuation of physiotherapy after surgery	Physical therapy: soft tissue stretching and strengthening (hip flexor stretching, soft tissue mobilization and joint mobilization); a muscle energy technique by Magee, acupuncture, (with no changes in symptoms); home exercise program (after surgery): basic postsurgical lower extremity ROM exercises followed by a progressive quadriceps, hamstring, adductor/ abductor strengthening program	ESWT
**Symptoms**	Foot pain	Bilateral hip pain, mobility restriction, secondary bilateral knee pain	Low back pain	Foot pain, mobility restriction, lower limb weakness	Hip pain and groin, hip snapping	Low back pain	Posterior knee pain	Medial ankle pain	Chronic hip and groin pain	Midfoot pain
**Injury**	Stress factures of the base of the metatarsal bones (II and III)	Bilateral hip pain and restriction	Low back pain	Condition after os trigonum excision	Acetabular labral tear	Low back pain	Posterior knee pain	Flexor hallucis longus (FHL) tendinopathy	Hip disfunction (unknown etiology)	Metatarsal fracture
**Gender**	F: 10; M: 9	F: 1	Not given	F: 1	F: 1	Not given	F: 1	F: 1	F: 1	F: 1
**Mean age**	16,4 (+/- 1,5)	14 years	12,7 (+/- 2,2); 13,7 (+/- 2,9)	15 years	29 years	11-18 years	16 years	17 years	18 years	29 years
**Sample size**	19	1	Intervention 30; Comparator 32	1	1	5	1	1	1	1
**Country**	Italy	New Zealand	Hungary	USA	USA	USA	USA	USA	USA	UK
**Study**	**Albisetti [27]**	**Baigent [29]**	**Kovácsné [32]**	**Filipa [30]**	**Kho-Summers [34]**	**Kline [33]**	**Mason [35]**	**Porter [36]**	**Quarrier [31]**	**Silk [28]**

Abbreviations: CT—computed tomography, DFOS—Dance functional outcome survey, EMF—electromagnetic fields, ESWT—external shock wave therapy, F–female, FSL squat—full single leg squat, LL-test—leg lowering test, M–male, MRI—magnetic resonance imaging, NRS—Numerical Rating Scale, PedsQL—The Pediatric Quality of Life Inventory, PSFS—the Patient Specific Functional Scale, ROM—range of motion, RTD—Return-To–Dance, SLR—straight leg rising, US—ultrasound therapy, USG—ultrasound scan, VAS—Visual Analogue Scale, X-ray—radiography

### Participant characteristics

The total number of participants in all the studies was 83. The number of subjects ranged from 1 to 62, aged 11–29. As two studies failed to provide information about the sex of the subjects [32, 33], the proportion of male and female subjects could not be determined. Pain and dysfunctions concerned the feet [27, 28, 30, 36], hips [29, 31, 34], lumbosacral spine [32, 33] and knees [35].

### Type of intervention

Even though all studies were concerned with the use of physiotherapy in the treatment of injuries in ballet dancers, variability with respect to both the type of intervention and the mode of application thereof was very much noticeable. Silk et al. investigated one intervention, while the others assessed several interventions each, which makes it difficult to determine causality. The interventions comprised shockwave therapy (ESWT) [27, 28], manual therapy [29–31], stability training for the lumbosacral spine [32, 33], a home exercise program following prior instruction as an adjunct to therapy [31, 33–35] as well as heat therapy [29, 30] and dry needling and acupuncture [31, 35].

### Outcome measures (OMs)

The types of outcome measures for assessing the effectiveness of physiotherapy interventions varied between the studies with regard to the nature of dysfunction and site of the problem. Both subjective and objective measures were used, including measures of pain intensity (VAS, NRS), muscle length tests (SLR, Thomas test, Ober test), diagnostic special test (Patrick), hypermobility (Beighton), physical assessment and posture and gait analysis. Imaging studies (MRI, x-ray, CT, ultrasound) were also used and mood was assessed with the Pediatric Quality of Life Inventory (PedsQL). Moreover, DFOS (Dance functional outcome survey) and PSFS (the Patient Specific Functional Scale) questionnaires were used.

Assessment time points also varied: medium term outcomes were assessed in all studies and short-term outcomes were assessed only by Mason et al. In the studies of Silk et al. and Quarrier et al., the desired outcomes were obtained at the 1-year and 6-year marks. Adverse effects of the physiotherapy intervention were not reported in any of the studies. The range of outcome domains and measures in specific studies is presented in Table 2.

**Table 2:** The range of outcome domains and measures in specific studies.

**Outcome Domain and Outcome Measures**	**Structural condition**	**USG**								✓		
		**CT**	✓									✓
		**X-ray**	✓	✓		✓			✓	✓	✓	✓
		**MRI**	✓			✓			✓	✓		
	**Posture and gait analysis**			✓	✓				✓	✓		
	**Questionnaires**	**PedsQL**				✓						
		**PSFS**						✓				
		**DFOS**				✓						
	**Physical assessment**	**Manual muscle tests**				✓					✓	
		**Deep squat**							✓			
		**Balance and proprioception tests**				✓						
		**FSL squat**							✓			
		**Sahrmann**					✓					
		**Core-tests**			✓			✓				
		**RTD**				✓						
		**LL-test**			✓							
		**ROM**		✓		✓		✓	✓	✓	✓	
	**Diagnostic special test**	**Patrick**									✓	
	**Muscle length tests**	**Ober**							✓		✓	
		**Thomas**							✓		✓	
		**SLR**					✓	✓	✓			
	**Hypermobility**	**Beighton**				✓						
	**Pain**	**N/G**	✓							✓	✓	✓
		**NRS**		✓		✓	✓	✓	✓			
		**VAS**			✓							
**Study**			**Albisetti [27]**	**Baigent [29]**	**Kovácsné [32]**	**Filipa [30]**	**Kho-Summers [34]**	**Kline [33]**	**Mason [35]**	**Porter [36]**	**Quarrier [31]**	**Silk [28]**

Abbreviations: CT—computed tomography, DFOS—Dance functional outcome survey, EMF—electromagnetic fields, ESWT—external shock wave therapy, F–female, FSL squat—full single leg squat, LL-test—leg lowering test, M–male, MRI—magnetic resonance imaging, NRS—Numerical Rating Scale, PedsQL—The Pediatric Quality of Life Inventory, PSFS—the Patient Specific Functional Scale, ROM—range of motion, RTD—Return-To–Dance, SLR—straight leg rising, US—ultrasound therapy, USG—ultrasound scan, VAS—Visual Analogue Scale, X-ray—radiography

#### Pain

Although pain was the dominant reason for a physiotherapy intervention in all dancers examined, 6 out of the 10 studies employed a subjective pain intensity scale (VAS [32] and NRS [29, 30, 33–35]), with the remaining studies failing to specify the measurement tool. All 10 studies stated that the physiotherapy intervention had produced a positive effect as the dancers were able to resume the practice of ballet pain-free following various time intervals from the beginning of the intervention.

#### Range of motion

In order to test the patients’ functional status, the ROM of selected joints was measured in six studies depending on the site of the dysfunction [29, 30, 31, 33, 35, 36]. Kline et al. employed a goniometer for that purpose, and the remaining studies did not specify the measurement tool. ROM was assessed in lower limb joints (hip, knee, foot), with only one study noting an unrestricted pain-free ROM [35] before the intervention. The physiotherapy produced the positive effect of regaining unrestricted pain-free ROM in the joints tested.

#### Physical assessment

Physical function was assessed with various tests before and after physiotherapy. Tests which produced a positive result before the intervention were repeated after the intervention. Improvement was noted in the SLR (straight leg raise) test in patients with hip and lower back pain [33, 34] and in core tests in patients with chronic low back pain [32, 33]. Two studies included the Thomas and Ober tests, which were negative [31, 35]. The Patrick test, leg lowering test, single leg squat and deep squat tests were also performed, as were tests such as return-to-dance and a movement system examination by Sahrmann. All tests demonstrated desired effects post-intervention.

#### Posture and gait analysis

Four studies investigated the dancers’ posture [29, 32, 35, 36] and gait [29, 35, 36]. To this end, a Camera Nicon Cool PIX L21 was used in the study of patients with low back pain [32], and the results were subjected to a statistical analysis. In three studies, this assessment revealed abnormalities that improved after the intervention [29, 32, 35].

#### Quality of life

Filipa et al. [30] employed a standardized questionnaire used for assessing quality of life with health in the paediatric population (PedsQL, the Pediatric Quality of Life Inventory). A baseline assessment of a 15-year-old female dancer with pain in the area of the left ankle joint (status post removal of os trigonum) was 68 (75%), improving to 100% post-intervention, indicating a reduction in pain-related depressive symbols. This was the only study out of the 10 which assessed dancers’ quality of life.

#### Structural status (imaging studies)

Imaging studies were performed in 8 out of the 10 studies [27–32, 35, 36]. They were often administered as part of diagnostic work-up, but were repeated in four studies in order to assess the effectiveness of the physiotherapy intervention. MRI was the only tool, apart from a subjective pain rating scale, to assess the effectiveness of ESWT in dancers with metatarsal fractures [27]. Improvement was noted in 17 of the 18 dancers after 3–5 weeks of therapy. One patient was treated with ultrasound therapy and EMF (electromagnetic fields), as the MRI showed a fracture line going through the metatarsal cartilage plate, and changes were additionally assessed with CT. Similar findings were reported by Silk et al. [28] in a female patient following a metatarsal fracture, with positive effects obtained with ESWT. Changes were assessed with conventional radiographs and CT before and on completion of a cycle of physiotherapy.

### Summary of results

The results of all 10 studies are summarized in 6 domains in Table 3. The results suggest that physiotherapy exerted a positive influence in all 6 domains. The evidence indicates that physiotherapy interventions reduced the signs and symptoms and improved function. Considering the small number of studies, these findings are encouraging, especially with regard to pain reduction and improvement in ROM and in physical functions. Despite those promising effects, caution is required when interpreting these results in view of methodological shortcomings, including the lack of statistical significance in most studies (9/10).

**Table 3:** The results of all 10 studies in 6 domains.

**Effect of physiotherapy interventions for the management of**	**Structural condition**	**USG**								(?)		
		**CT**	(?)									(+) ↑
		**X-ray**	(=) = 10; (?) = 9	(=)		(?)			(?)	(=)	(=)	(+) ↑
		**MRI**	(+) ↑			(?)			(?)	(?)		
	**Posture and gait analysis**			(+) ↑	(+) ↑				(+) ↑	**(=)9=0**		
	**Questionnaires**	**PedsQL**				(+) ↑						
		**DFOS**				(+) ↑						
		**PSFS**						(+) ↓				
	**Physical assessment**	**Manual muscle tests**				(+) ↑					(+) ↑	
		**Deep squat**							(+) ↑			
		**Balance and proprioception tests**				(+) ↑						
		**FSL squat**							(+) ↑			
		**Sahrmann**					(+) ↑					
		**Core-tests**			(+) ↑ *			(+) ↑				
		**RTD**				(+) ↑						
		**LL-test**			(+) ↓ *							
		**ROM**		(+) ↑		(+) ↑		(+) ↑	(+) ↑	(+) ↑	(+) ↑	
	**Diagnostic special test**	**Patrick**									(?)	
	**Muscle length tests**	**Ober**							(=)		(=)	
		**Thomas**							(=)		(=)	
		**SLR**					(+) ↑	(+) ↑	**(=)**			
	**Hypermobility**	**Beighton**				(?)						
	**Pain**	**N/G**	(+) ↓							(+) ↓	(+) ↓	(+) ↓
		**NRS**		(+) ↓		(+) ↓	(+) ↓	(+) ↓	(+) ↓			
		**VAS**			(+) ↓ *							
**Study**			**Albisetti [27]**	**Baigent [29]**	**Kovácsné [32]**	**Filipa [30]**	**Kho-Summers [34]**	**Kline [33]**	**Mason [35]**	**Porter [36]**	**Quarrier [31]**	**Silk [28]**

Abbreviations: CT—computed tomography, DFOS—Dance functional outcome survey, EMF—electromagnetic fields, ESWT—external shock wave therapy, F–female, FSL squat—full single leg squat, LL-test—leg lowering test, M–male, MRI—magnetic resonance imaging, NRS—Numerical Rating Scale, PedsQL—The Pediatric Quality of Life Inventory, PSFS—the Patient Specific Functional Scale, ROM—range of motion, RTD—Return-To–Dance, SLR—straight leg rising, US—ultrasound therapy, USG—ultrasound scan, VAS—Visual Analogue Scale, X-ray–radiography

### NHMRC FORM framework

A summary of the results with the NHMRC FORM framework is presented in Table 4. Despite the positive results, methodological concerns regarding the evidence base lowered the overall level of recommendations. While these results may be helpful in physiotherapy of dancers, the recommendations should be implemented with caution.

**Table 4:** NHMRS FORM framework.

Component	Grade	Comments
1. Evidence base	*D–Poor*	Quantiti: 10 studies
		Participants: 83 ballet dancers with musculoskeletal pain
	*Level IV studies*, *or level I to III studies with high risk of bias*	Level II: 0 studies
		Level III-2: 1 study
		Level III-3: 1 study
		Level IV: 8 studies
2. Consistency	*C–Satisfactory*	Findings consistent
	*Some inconsistency reflecting genuine uncertainty around clinical question*	Multiple study designs
		Heterogeneous interventions
		Varied population–injury type, age
		Varied outcome measures and time point measurements
3. Clinical impact	*D–Poor*	Consistent findings for outcomes: in particular pain
	*Slight*	
		Only one study has statistical significance
		The clinical significance should be approached with caution
		No adverse effects reported
4. Generalisability	*B–Good*	Population of studies is similar to the target
	*Population/s studied in the body of evidence are similar to the target population for the guideline*	
		Age range: 11–29 years
		Despite various types of injuries and interventions, symptoms in the entire population were associated with ballet dance training
		Studies conducted in five different countries that have different health care contexts
5. Grade of recommendations	*D—poor*	These studies had low evidence and were of moderate methodological quality.
	*The evidence is weak*, *so the recommendations should be used with caution*	
		Although overall there were positive results, the current evidence base is not homogeneous in terms of diagnosis, interventions delivered, and parameters and results measured for ballet dancers

## Discussion

The present systematic review aimed to investigate scientific evidence regarding physiotherapy interventions in ballet dancers. It is the first systematic review to present physiotherapeutic management in dancers. The evidence base was rather modest, with 10 studies representing different research projects. The summary of the results indicates that physiotherapy interventions may exert a positive influence in several domains, such as pain, ROM, functional status, posture, gait or quality of life. Consistent evidence in favor of effectiveness of physiotherapy was particularly demonstrated with regard to pain reduction, which indicates that physiotherapy interventions may be instrumental in the return of dancers to practice and performances. Despite these positive findings, the results need to be interpreted with caution in view of methodological limitations and non-homogeneity of the evidence base.

Physiotherapy interventions in ballet dancers were associated with positive effects in several areas as demonstrated by both positive and negative measurements. This revealed a potential for using physiotherapy in this group of patients.

There is evidence confirming analgesic effectiveness of similar interventions in physically active populations, for example with ESWT [37, 38]. Hides et al. confirmed the effectiveness of stability training for the lumbosacral spine in athletes [39]. However, other authors of systematic reviews have objected to formulating strong conclusions about this type of training as an isolated intervention for improving sports results, including pain reduction and reducing recovery time following an injury [40, 41]. It has also been reported that dry needling produced the desired effects in the treatment of knee pain both as a sole intervention in athletes with patellofemoral knee syndrome [42, 43] and in conjunction with manual therapy and exercise in patients with osteoarthritis [44].

The effectiveness of physiotherapy in dancers was assessed with various functional tests and, often, ROM measurements. The following tests are often used for assessing the functional status of the lower limbs and lumbosacral spine owing to ease of administration, simplicity and reproducibility: SLR, Patrick test, Thomas test, single leg squat test and others [45–48]. In view of the aesthetic and technical demands of dance, associated with extreme ROM, Filipa et al. used the Beighton scale to identify joint hypermobility syndrome (JHS) [30]. Other studies have considered JHS a risk factor for experiencing pain in children and adults [49, 50]. However, the evidence that this tendency is also present in dancers in not equivocal [51, 52]. There are functional assessment scales designed with dancers in mind, such as the Dance Functional Outcome Survey (DFOS), which was used in one study [30]. Other authors have also used this tool to assess the effectiveness of comprehensive rehabilitation of a modern female dancer with metatarsal instability, achieving an improvement from 11% to 90% [53].

Filipa et al. reported improved mood on completion of the physiotherapy intervention [30], using the standardized PedsQL questionnaire as a tool. This was probably due to the positive influence of pain reduction and resumption of dance practice. Earlier studies had reported higher levels of burnout in dancers [54] and athletes [55, 56], who suffered physical and emotional exhaustion, compared to injury-free individuals. Reduced quality of life was also reported in injured athletes [57]. This confirms a significant negative effect of pain on depressive mood and increased risk of burnout in physically active individuals. Depressed mood in dancers may be associated with excessive physical training, which, combined with other external factors, may lead to injuries and overload syndromes, which may produce a general deterioration of health and well-being in the dancers [58]. Furthermore, the break from practice in injured athletes may reduce their quality of life. Adequate injury perception by physically active individuals would facilitate evidence-based treatment and physiotherapy strategies targeting the physical and psychosocial aspects of health [59]. Consequently, this systematic review can prove very useful in planning effective physiotherapy interventions in dancers.

## Limitations

Even though the present paper is based on the best practices for systematic reviews (PRISMA), it is not free of limitations. The review was based on electronic data bases, implementing secondary search strategies. As a result, certain studies may have remained unidentified and excluded from the review. The exclusion of non-English publications was another limitation.

Ultimately, a total of 10 publications meeting the inclusion criteria were qualified, which is a modest evidence base, although the findings were actually consistently positive. At the same time, there were certain concerns and limitations as regards the methodological quality of the studies reviewed. 80% of the studies were case reports or case series, and, therefore, the provision of a rationale for the sample size and statistical analysis might turn out to be unnecessary or impossible. Accordingly, there are no grounds for extrapolating these results to the entire population of ballet dancers. Furthermore, in some of the studies, physiotherapy procedures were supplemented by other interventions, such as surgery.

## Conclusions

The positive effects of physiotherapeutic interventions have highlighted the potential role of physiotherapy for ballet dancers after injuries, which are an important health problem in this specific group of patients. Due to the small amount of evidence confirming the effectiveness of physiotherapeutic interventions in ballet dancers after injuries and methodological uncertainties, it is recommended to improve the quality of prospective studies. The use of more standard results with long observation periods would help to identify potential therapeutic effects in ballet dancers.

## Practical implications

There is evidence to support the use of physiotherapy interventions in injured ballet dancers. Physiotherapy exerted a positive influence in several domains, including pain, ROM and functional status. However, while physiotherapy may be considered as an option for managing injuries in ballet dancers, caution should be exercised while these recommendations are implemented on account of methodological concerns regarding the existing evidence base.

## Supporting information

S1 Fig PRISMA 2009 flow diagram. (DOC)

S2 Fig Items on Modified McMaster Critical Review form. (TIF)

S1 File PRISMA 2009 checklist. (DOC)

S2 File Search protocols. (DOCX)

## References

[1] Batista CG, Martins EO. The prevalence of pain in classical ballet dancers. J Health Sci Inst. 2010;28(1):47–49.

[2] Twitchett EA, Koutedakis Y, Wyon MA. Physiological fitness and professional classical ballet performance: a brief review. J Strength Cond Res. 2009; 23:2732–2740. doi: 10.1519/JSC.0b013e3181bc1749 19910802

[3] Allen N, Nevill A, Brooks J, Koutedakis Y, Wyon M. Ballet Injuries: injury incidence and severity over 1 year. J Orthop Sports Phys Ther. 2012; 42(9):781–790 doi: 10.2519/jospt.2012.3893 22814244

[4] Drężewska M, Śliwiński Z. Lumbosacral pain in balet school students. Pilot study. Ortop Traumatol Rehabil 2013; 15(2):149–58. doi: 10.5604/15093492.1041451 23652535

[5] Costa MS, Ferreira AS, Orsini M, Silva EB, Felicio LR. Characteristics and prevalence of musculoskeletal injury in professional and non-professional ballet dancers. Braz J Phys Ther. 2016; 20(2): 166–175. doi: 10.1590/bjpt-rbf.2014.0142 26786085 PMC4900039

[6] Zaletel P, Sekulic D, Zenic N, Esco MR, Sajber D, Kondric M. The association between body-built and injury occurrence in pre-professional ballet dancers—Separated analysis for the injured body-locations. Int. J. Occup. Med. Environ. Health. 2017; 30:151–159. doi: 10.13075/ijomeh.1896.00818 28220914

[7] Hendry D, Campbell A, Ng L, Grisbrook TL, Hopper DM. Effect of Mulligan’s and Kinesio knee taping on adolescent ballet dancers knee and hip biomechanics during landing. Scand J Med Sci Sports. 2015; 25(6):888–96. doi: 10.1111/sms.12302 25091570

[8] Liederbach M, Kremenic IJ, Orishimo KF, Pappas E, Hagins M. Comparison of landing biomechanics between male and female dancers and athletes, part 2: influence of fatigue and implications for anterior cruciate ligament injury. Am J Sports Med. 2014; 42:1089–1095. doi: 10.1177/0363546514524525 24595401

[9] Henn ED, Smith T, Ambegaonkar JP, Wyon M. Low back pain and injury in ballet, modern and hip-hop dancers: a systematic review. Int J Sports Phys Ther 2020; 15(5): 671–687. doi: 10.26603/ijspt20200671 33110686 PMC7566832

[10] Smith PJ, Gerrie BJ, Varner KE, McCulloch PC, Lintner DM, Harris JD. Incidence and Prevalence of Musculoskeletal Injury in Ballet: A Systematic Review. Orthop J Sports Med. 2015; 3(7):2325967115592621. doi: 10.1177/2325967115592621 26673541 PMC4622328

[11] Steinberg N., Aujla I., Zeev A., Redding E. Injuries among talented young dancers: Findings from the U.K. Centres for Advanced Training. Int. J. Sports Med. 2014;35:238–244. doi: 10.1055/s-0033-1349843 23900897

[12] Biernacki J.L., Stracciolini A., Fraser J., Lyle J.M., Sugimoto D. Risk factors for lower-extremity injuries in female ballet dancers: A systematic review. Clin. J. Sport Med. 2018 doi: 10.1097/JSM.0000000000000707 30589745

[13] Twitchett E., Brodrick A., Nevill A.M., Koutedakis Y., Angioi M., Wyon M. Does physical fitness affect injury occurrence and time loss due to injury in elite vocational ballet students? J. Dance Med. Sci. 2010; 14:26–31. 20214852

[14] Roussel N., De Kooning M., Schutt A., Mottram S., Truijen S., Nijs J., et al. Motor control and low back pain in dancers. Int. J. Sports Med. 2013; 34:138–143. doi: 10.1055/s-0032-1321722 22960991

[15] Alderson JA, Hopper LS, Elliot BC, Ackland T. Risk factors for lower back injury in male dancers performing ballet lifts. J Dance Med Sci 2009; 13(3):83–9. 19754984

[16] Kenny SJ, Whittaker JL, Emery CA. Risk factors for musculoskeletal injury in preprofessional dancers: a systematic review. Br J Sports Med 2016;50(16):997–1003. doi: 10.1136/bjsports-2015-095121 26626269

[17] Nihal A, Rose DJ, Trepman E. Arthroscopic Treatment of Anterior Ankle Impingement Syndrome in Dancers. Foot Ankle Int. 2005; 26(11):908–12. doi: 10.1177/107110070502601102 16309602

[18] Russell JA, Kruse DW, Koutedakis Y, Mcewan IM, Wyon MA. Pathoanatomy of posterior ankle impingement in ballet dancers. Clin. Anat. 2010; 23:613–621. doi: 10.1002/ca.20991 20821398

[19] Moez SB, Roche A, Brodrick A, Williams RL, Calder JD. Posterior Endoscopic Excision of Os Trigonum in Professional National Ballet Dancers. J Foot Ankle Surg Sep-Oct 2016;55(5):927–30. doi: 10.1053/j.jfas.2016.04.006 27289219

[20] Amari R, Sakai T, Katoh S, Sairyo K, Higashino K, Tachibana K, et al. Fresh stress fractures of lumbar pedicles in an adolescent male ballet dancer: Case report and literature review. Arch Orthop Trauma Surg. 2009; 129(3):397–401. doi: 10.1007/s00402-008-0685-8 18607611

[21] Martinez SF, Murphy GA. Tibial stress fracture in a male ballet dancer: a case report. Am J Sports Med. 2005; 33(1):124–30. doi: 10.1177/0363546504266479 15611009

[22] Prisk V, Hamilton WG. Stress Fracture of the First Rib in Weight-Trained Dancers. Am J Sports Med. 2008; 36(12):2444–7. doi: 10.1177/0363546508326710 18948414

[23] Miyamoto RG, Dhotar HS, Rose DJ, Egol K. Surgical Treatment of Refractory Tibial Stress Fractures in Elite Dancers: A Case Series. Am J Sports Med. 2009; 37(6):1150–4. doi: 10.1177/0363546508330973 19293326

[24] Weber AE, Bedi A, Tibor LM, Zaltz I, Larson CM. The Hyperflexible Hip: Managing Hip Pain in the Dancer and Gymnast. Sports Health. 2015; 7(4):346–58. doi: 10.1177/1941738114532431 26137181 PMC4481673

[25] Nolton EC, Ambegaonkar JP. Recognizing and Managing Snapping Hip Syndrome in Dancers. Med Probl Perform Art. 2018; 33(4):286–291. doi: 10.21091/mppa.2018.4042 30508831

[26] Law M, Stewart D, Pollock N, Letts L, Bosch J, Westmorland M. Critical Review Form–Quantitative Studies. Occupational Therapy Evidence-Based Practice Research Group. Canada: McMaster University; 1998.

[27] Albisetti W, Perugia D, De Bartolomeo O, Tagliabue L, Camerucci E, Calori GM. Stress Fractures of the Base of the Metatarsal Bones in Young Trainee Ballet Dancers. Int Orthop. 2010; 34(1):51–5. doi: 10.1007/s00264-009-0784-3 19415273 PMC2899256

[28] Silk ZM, Alhuwaila RS, Calder JD. Low-energy Extracorporeal Shock Wave Therapy to Treat Lesser Metatarsal Fracture Nonunion: Case Report. Foot Ankle Int. 2012; 33(12):1128–32. doi: 10.3113/FAI.2012.1128 23199865

[29] Baigent L. Chiropractic Management of a 14-Year-Old Ballet Dancer with Bilateral Hip Pain and Restriction. Journal of Clinical Chiropractic Pediatrics. 2013; 14(1):1103–09.

[30] Filipa A, Barton K. Physical Therapy Rehabilitation of An Adolescent Pre-Professional Dancer Following Os Trigonum Excision: A Case Report. J Orthop Sports Phys Ther. 2018; 48(3):194–203. doi: 10.2519/jospt.2018.7508 29113569

[31] Quarrier NF, Wightman AB. A Ballet Dancer With Chronic Hip Pain Due to a Lesser Trochanter Bony Avulsion: The Challenge of a Differential Diagnosis. J Orthop Sports Phys Ther. 1998; 28(3):168–73. doi: 10.2519/jospt.1998.28.3.168 9742474

[32] Kovácsné BV, Szilágyi B, Kiss G, Leidecker E, Ács P, Oláh A, et al. Application and examination of the efficiency of a core stability training program among dancers. European Journal of Integrative Medicine 2016; 8(2):3–7.

[33] Kline JB, Krauss JR, Maher SF, Qu X. Core Strength Training Using a Combination of Home Exercises and a Dynamic Sling System for the Management of Low Back Pain in Pre-professional Ballet Dancers: A Case Series. J Dance Med Sci. 2013; 17(1):24–33. doi: 10.12678/1089-313x.17.1.24 23498354

[34] Khoo-Summers L, Bloom NJ. Examination and treatment of a professional ballet dancer with a suspected acetabular labral tear: A case report. Man Ther. 2015; 20(4):623–9. doi: 10.1016/j.math.2015.01.015 25725589

[35] Mason JS, Tansey KA, Westrick RB. Treatment of Subacute Posterior Knee Pain in an Adolescent Ballet Dancer Utilizing Trigger Point Dry Needling: A Case Report. Int J Sports Phys Ther. 2014; 9(1):116–24. 24567862 PMC3924615

[36] Porter EB, Dubois MS, Raasch WG. A 17-year-old Ballet Dancer With Medial Ankle Pain. Curr Sports Med Rep. 2010; 9(5):290–1. doi: 10.1249/JSR.0b013e3181f2731e 20827094

[37] Vulpiani MC, Vetrano M, Savoia V, Pangrazio ED, Trischitta D, Ferretti A. Jumper’s Knee Treatment With Extracorporeal Shock Wave Therapy: A Long-Term Follow-Up Observational Study. J Sports Med Phys Fitness. 2007; 47(3):323–8. 17641600

[38] Moen MH, Rayer S, Schipper M, Schmikli S, Weir A, Tol JL, et al. Shockwave Treatment for Medial Tibial Stress Syndrome in Athletes; A Prospective Controlled Study. Br J Sports Med. 2012; 46(4):253–7. doi: 10.1136/bjsm.2010.081992 21393260

[39] Hides J, Stanton W, McMahon S, Sims K, Richardson C. Effect of Stabilization Training On Multifidus Muscle Cross-sectional Area Among Young Elite Cricketers With Low Back Pain. J Orthop Sports Phys Ther. 2008; 38(3):101–8. doi: 10.2519/jospt.2008.2658 18349481

[40] Haugen T, Haugvad L, Røstad V. Effects of Core-Stability Training on Performance and Injuries in Competitive Athletes. Sportscience. 2016; 20:1–7.

[41] Stuber K, Bruno P, Sajko S, Hayden J. Core Stability Exercises for Low Back Pain in Athletes: A Systematic Review of the Literature. Clin J Sport Med. 2014; 24(6):448–56. doi: 10.1097/JSM.0000000000000081 24662572

[42] Zarei Z, Bervis S, Piroozi S, Motealleh A. Added Value of Gluteus Medius and Quadratus Lumborum Dry Needling in Improving Knee Pain and Function in Female Athletes With Patellofemoral Pain Syndrome: A Randomized Clinical Trial. Arch Phys Med Rehabil. 2020; 101(2):265–274. doi: 10.1016/j.apmr.2019.07.009 31465756

[43] Sutlive TG, Golden A, King K, Morris WB, Morrison JE, Moore JH, et al. Short-term effects of trigger point dry needling on pain and disability in subjects with patellofemoral pain syndrome. Int J Sports Phys Ther. 2018 Jun; 13(3): 462–473. 30038832 PMC6044598

[44] Dunning J, Butts R, Young I, Mourad F, Galante V, Bliton P, et al. Periosteal Electrical Dry Needling as an Adjunct to Exercise and Manual Therapy for Knee Osteoarthritis. Clin J Pain. 2018; 34(12):1149–1158. doi: 10.1097/AJP.0000000000000634 29864043 PMC6250299

[45] Gatha P, Khushboo B, Smita K, Amrutkuwar P, Vishnupriya D, Prachi J. Efectiveness of myofascial release, static stretching and neutral tissue mobilization on hamstring flexibility in athletes. Indian Journal of Public Health Research & Development 2019; 10(4):6–11.

[46] Fournier-Farley C, Lamontagne M, Gendron P, Gagnon DH. Determinants of Return to Play After the Nonoperative Management of Hamstring Injuries in Athletes A Systematic Review. Am J Sports Med. 2016; 44(8):2166–72. doi: 10.1177/0363546515617472 26672025

[47] Vigotsky AD, Lehman GJ, Contreras B, Beardsley C, Chung B, Feser EH. Acute Effects of Anterior Thigh Foam Rolling on Hip Angle, Knee Angle, and Rectus Femoris Length in the Modified Thomas Test. PeerJ 2015; 24(3). doi: 10.7717/peerj.1281 26421244 PMC4586805

[48] Halabchi F, Abolhasani M, Mirshahi M, Alizadeh Z. Patellofemoral pain in athletes: clinical perspectives. Open Access J Sports Med. 2017; 8:189–203. doi: 10.2147/OAJSM.S127359 29070955 PMC5640415

[49] Sohrbeck-Nøhr O, Kristensen JH, Boyle E, Remvig L, and Juul-Kristensen B. Generalized joint hypermobility in childhood is a possible risk for the development of joint pain in adolescence: a cohort study. BMC Pediatr. 2014; 14: 302. doi: 10.1186/s12887-014-0302-7 25492414 PMC4305244

[50] Pacey V, Nicholson LL, Adams RD, Munn J, Munns CF. Generalized Joint Hypermobility and Risk of Lower Limb Joint Injury During Sport: A Systematic Review With Meta-Analysis. Am J Sports Med. 2010; 38(7):1487–97. doi: 10.1177/0363546510364838 20601606

[51] Sanches SB, Oliveira GM, Osório FL, Crippa JAS, Martín-Santos R. Hypermobility and joint hypermobility syndrome in Brazilian students and teachers of ballet dance. Rheumatology International 2015; 35:741–747. doi: 10.1007/s00296-014-3127-7 25218649

[52] Bronner S, Bauer NG. Risk factors for musculoskeletal injury in elite pre-professional modern dancers: A prospective cohort prognostic study. Physical Therapy in Sport. 2018; 31:42–51. doi: 10.1016/j.ptsp.2018.01.008 29597115

[53] Ojofeitimi S, Bronner S, Becica L. Conservative Management of Second Metatarsophalangeal Joint Instability in a Professional Dancer: A Case Report. J Orthop Sports Phys Ther. 2016; 46(2):114–123. doi: 10.2519/jospt.2016.5824 26755404

[54] Drężewska M, Śliwiński Z, Śliwiński GE. Perfectionism and Burnout in Sport and Dance. Phys Med Rehab Kuror. 2020; 30(03):135–140.

[55] Moen F, Myhre K, Klöckner CA, Gausen K, Sandbakk Ø. Physical, Affective and Psychological determinants of Athlete Burnout. Sport Journal 2017; 4(27): 1–14.

[56] Hughes P. Association Between Athlete Burnout and Athletic Injury. University of North Carolina at Chapel Hill Graduate School 2014; doi: 10.17615/ymb8-gp39

[57] McGuine TA, Winterstein AP, Carr K, Hetzel S. Changes in Health-Related Quality of Life and Knee Function After Knee Injury in Young Female Athletes. Orthopaedic Journal of Sports Medicine. Orthop J Sports Med. 2014 Apr; 2(4): 2325967114530988. doi: 10.1177/2325967114530988 26535324 PMC4555590

[58] Koutedakis Y. “Burnout” in Dance The Physiological Viewpoint. The Journal of Dance Medicine & Science. 2000; 4(4):122–127.

[59] Houston MN, Hoch JM, Van Lunen BL, Hoch MC. The Impact of Injury on Health-Related Quality of Life in College Athletes. Journal of Sport Rehabilitation. 2017; 26(5):365–375. doi: 10.1123/jsr.2016-0011 27632873

