# Supplementary material for: Effectiveness of physiotherapy interventions for injury in ballet dancers: A systematic review
Source: PLoS One. 2021 Jun 24;16(6):e0253437. doi: 10.1371/journal.pone.0253437 (PMC8224967; doi:10.1371/journal.pone.0253437)
Supplement: S1 Fig — (DOC) [file pone.0253437.s001.doc]

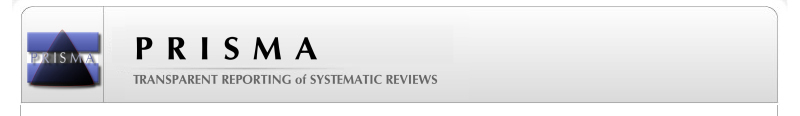
**PRISMA 2009 Flow Diagram**

PubMed

304 Citations

Embase Ovid

214 Citations

Cochrane

49 Citations

Medline

62 Citations

Google Scholar

55 Citations

PEDro

3 Citations

**Screening**

**Included**

**Eligibility**

**Identification**

Records identified through database searching
(n = 687)

Records after duplicates removed
(n = 482)

Records screened
(n = 482)

Records excluded
(n = 404)

Full-text articles assessed for eligibility
(n = 78)

Full-text articles excluded, with reasons
(n = 68):

35 Wrong patient poplation

24 Wrong study design

9 Wrong intervention

Studies included in qualitative synthesis
(n = 10)

Duplicates removed

(n = 205)
