# Supplementary figures and images for: Effectiveness of physiotherapy interventions for injury in ballet dancers: A systematic review
Source: PLoS One. 2021 Jun 24;16(6):e0253437. doi: 10.1371/journal.pone.0253437 (PMC8224967; doi:10.1371/journal.pone.0253437)

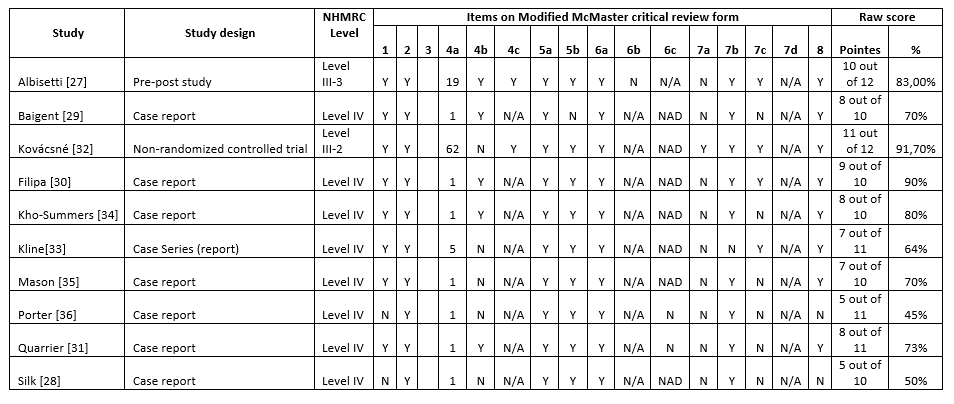

Supplement: S2 Fig — (TIF) [file pone.0253437.s002.tif]
