# Supplementary material for: Effectiveness of physiotherapy interventions for injury in ballet dancers: A systematic review
Source: PLoS One. 2021 Jun 24;16(6):e0253437. doi: 10.1371/journal.pone.0253437 (PMC8224967; doi:10.1371/journal.pone.0253437)
Supplement: S2 File — (DOCX) [file pone.0253437.s004.docx]

Search protocols

Pubmed:

Search: **((((dance therapy[MeSH Terms]) AND (ballet[MeSH Terms])) OR (ballet dancer)) OR (classical dancer)) AND (pain)**

(("dance therapy"[MeSH Terms] AND "dancing"[MeSH Terms]) OR (("balletic"[All Fields] OR "dancing"[MeSH Terms] OR "dancing"[All Fields] OR "ballet"[All Fields] OR "ballets"[All Fields]) AND ("dancer"[All Fields] OR "dancer s"[All Fields] OR "dancers"[All Fields])) OR (("classic"[All Fields] OR "classical"[All Fields] OR "classically"[All Fields] OR "classicals"[All Fields] OR "classics"[All Fields]) AND ("dancer"[All Fields] OR "dancer s"[All Fields] OR "dancers"[All Fields]))) AND ("pain"[MeSH Terms] OR "pain"[All Fields])

**Translations**

**dance therapy[MeSH Terms]:** "dance therapy"[MeSH Terms]

**ballet[MeSH Terms]:** "dancing"[MeSH Terms]

**ballet:** "balletic"[All Fields] OR "dancing"[MeSH Terms] OR "dancing"[All Fields] OR "ballet"[All Fields] OR "ballets"[All Fields]

**dancer:** "dancer"[All Fields] OR "dancer's"[All Fields] OR "dancers"[All Fields]

**classical:** "classic"[All Fields] OR "classical"[All Fields] OR "classically"[All Fields] OR "classicals"[All Fields] OR "classics"[All Fields]

**dancer:** "dancer"[All Fields] OR "dancer's"[All Fields] OR "dancers"[All Fields]

**pain:** "pain"[MeSH Terms] OR "pain"[All Fields]

**Google Scholar**

allintitle: ballet therapy OR treatment

**PEDro**

Search term: ballet

Subdiscipline: musculoskeletal

**Cochrane Library**

## ballet in Title Abstract Keyword OR ballet dancer in Title Abstract Keyword AND physical therapy in Title Abstract Keyword AND pain in Title Abstract Keyword

**Embase**

(**ballet**:ti,ab,kw OR **'ballet dancer'**:ti,ab,kw) AND **pain**:ti,ab,kw OR **'physical therapy'**:af

**Medline**

MH ballet OR TX ballet dancer AND AB ( physical therapy or physiotherapy or rehabilitation or physical treatment or exercise ) AND AB pain OR TX classical dancer
